# Bisphenol A-Related Effects on Bone Morphology and Biomechanical Properties in an Animal Model

**DOI:** 10.3390/toxics10020086

**Published:** 2022-02-14

**Authors:** Tobias Prasse, Ioannis Stratos, Anja Niehoff, Hildegard Christ, Vincent Heck, Carolin Meyer, Thomas Mittlmeier

**Affiliations:** 1 Department of Orthopedics and Trauma Surgery, Faculty of Medicine and University Hospital of Cologne, University of Cologne, Kerpener Str. 62, 50937 Cologne, Germany; vincent.heck@uk-koeln.de; 2 Department of Orthopedics, Würzburg University Medical Center, König-Ludwig-Haus, Brettreichstraße 11, 97074 Würzburg, Germany; i-stratos.klh@uni-wuerzburg.de; 3 Institute of Biomechanics and Orthopaedics, German Sport University Cologne, Am Sportpark Müngersdorf 6, 50933 Cologne, Germany; niehoff@dshs-koeln.de; 4 Cologne Center for Musculoskeletal Biomechanics, Faculty of Medicine and University Hospital of Cologne, Joseph-Stelzmann-Str. 9, 50931 Cologne, Germany; 5 Institute of Medical Statistics and Bioinformatics, University of Cologne, Robert-Koch-Straße 10, 50931 Cologne, Germany; hchrist@uni-koeln.de; 6 Center for Spinal Surgery, Helios Klinikum Bonn/Rhein-Sieg, Von-Hompesch-Straße 1, 53123 Bonn, Germany; caromeyerkoeln@gmail.com; 7 Department of Trauma, Hand and Reconstructive Surgery, Rostock University Medical Center, Schillingallee 35, 18057 Rostock, Germany; thomas.mittlmeier@med.uni-rostock.de

**Keywords:** bisphenol A, endocrine disruption, bone morphology, micro-computed tomography, mechanical property, three-point bending

## Abstract

Bisphenol A (BPA), which is contained in numerous plastic products, is known to act as an endocrine-disruptive, toxic, and carcinogenic chemical. This experimental series sought to determine the influence of BPA exposure on the femoral bone architecture and biomechanical properties of male and female Wistar rats. BPA was applied subcutaneously by using osmotic pumps. After 12 weeks, the bones were analyzed by micro-computed tomography (micro-CT) and a three-point bending test. Comparing the female low- and high-dose groups, a significantly greater marrow area (*p* = 0.047) was identified in the group exposed to a higher BPA concentration. In addition, the trabecular number tended to be higher in the female high-dose group when compared to the low-dose group (*p* > 0.05). The area moment of inertia also tended to be higher in the male high-dose group when compared to the male low-dose group (*p* > 0.05). Considering our results, BPA-related effects on the bone morphology in female Wistar rats are osteoanabolic after high-dose exposure, while, in male rats, a tendency toward negative effects on the bone morphology in terms of a reduced cross-sectional cortical area and total area could be demonstrated.

## 1. Introduction

Bisphenol A (BPA) is one of the most frequently produced chemical compounds worldwide, and it is a harmful substance for the human organism, which is why it is part of the American National Toxicology Program and the subject of numerous studies [1]. After the first synthesis of BPA in 1891 by Dianin, Dodds and Lawson outlined 45 years later that BPA had estrogenic effects [2,3,4]. Today, BPA is part of the synthesis of various plastic polymers, polycarbonates, and epoxy resins [5]. Thus, humans are in constant and close contact with this chemical, especially because BPA can be found in many food products through the wrapping and canning as part of the packaging process [5,6]. Lorber et al. estimated that the average amount of BPA ingested by adults per kg of body weight is between 0.03 and 0.07 μg per day and may be even higher in Asian countries [7,8].

BPA acts as an endocrine-disruptive chemical and has antiandrogenic effects. It binds to estrogen receptors (ER alpha and beta) and interacts with estrogen related pathways [9,10,11]. Furthermore, BPA-related toxic effects decrease sperm quality and reproductivity, and it can encourage the development of malignant diseases such as prostate and breast cancer, which is the subject of a controversial discussion [10,12,13,14,15,16]. In 2018, Chin et al. highlighted the effects of BPA and its derivates on bone in a review [17]. By affecting the endocrine system, BPA causes stress on osseous tissue, leading to cell death and DNA damage [18]. BPA inhibits osteoclast formation in vitro and induces apoptosis of both osteoblasts and osteoclasts by interfering with their differentiation [19]. Ovariectomized rats showed trabecular bone loss after BPA exposure, which could not be demonstrated in male rats, where the bone density was higher in BPA exposed groups. In contrast, bisphenol A diglycidyl ether (BADGE), a derivative of BPA, led to an increase in trabecular number in female and male rats, as well as to an increased bone density in female rats [17]. In addition, Li et al. concluded that BADGE improves bone quality in rat animal models and is osteoinductive in non-ovariectomized rats. There was no significant bone formation in ovariectomized rats.

The molecular mechanism underlying the decrease in bone density occurs via binding of BPA to the estrogen-dependent gamma-type receptors, according to a study by Thent et al. [20]. This binding reduces bone morphogenic protein 2 (BMP-2) formation and alkaline phosphatase activity [20]. BPA disrupts the bone metabolism via the receptor activator of NF-κB ligand (RANKL), apoptosis, and the Wnt/beta-catenin signaling pathway [20]. Thent et al. also described BPA exposure as resulting in a loss of bone mass due to decreased plasma calcium levels and inhibition of calcitonin secretion.

Facing these different and somewhat contradictory studies, our aim was to identify the effects of low- and high-dose BPA on the structure and the biomechanical properties of bone in growing male and female Wistar rats via a continuous application of BPA.

## 2. Materials and Methods

### 2.1. Experimental Setup

In this study, 36 Wistar rats that were 10 weeks old (Charles River Laboratories, Research Models and Services; Sulzfeld, Germany) were randomly assigned to one of three different groups: control (*n* = 12), low-dose BPA (*n* = 10), and high-dose BPA (*n* = 12). Thus, three groups each for male and female rats created six groups in total. The animals were housed in our local facility (Institute for Experimental Surgery; Rostock, Germany) under a 12 h day/night cycle and had ad libitum access to food and water. The rats were housed in a climatized room at a temperature of 22 °C and a relative humidity of 51%.

The daily dose in the low-dose group was 2.55 µg of BPA, while that in the high-dose group was 39 µg of BPA per day and rat. Transferring the low-dose (10.2 µg of BPA per kg bodyweight per day) and high-dose (156 µg of BPA per kg bodyweight per day) administration to an adult human with 70 kg body weight, this would be equal to 714 µg of BPA per day in the low-dose group and 10,920 µg of BPA per day in the high-dose group. The different dosages of BPA that were administered are based on the scientific opinion of the European Food Safety Authority (EFSA) in 2015. The rats weighed approximately 0.25 kg at the beginning of the experiments. The low-dose group reflects a dosage above the most recently defined tolerable daily intake (TDI) by the EFSA in 2015 (4 µg of BPA per kilogram bodyweight per day) [21]. BPA was dissolved in DMSO, which is ubiquitously used as a solvent [22]. The control group was exposed to DMSO only using osmotic pumps from the same manufacturer. The control group was not exposed to BPA.

The osmotic pumps were chosen in order to apply either BPA or the control solution evenly and with a constant flow rate. The manufacturer guaranteed constant application for 4 weeks for each pump. The osmotic pumps were used to achieve maximum consistency when administering BPA. The subcutaneous administration of BPA is known to have identical effects on the BPA plasma concentration to the oral route while being more practical [23,24,25].

**Figure 1:**
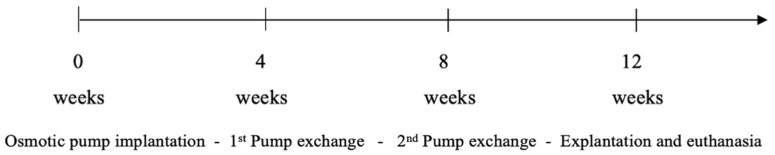
Timeline of the experimental setup.

During the 12 weeks of survival, the animals underwent two additional surgeries for an exchange of the pumps 4 and 8 weeks after initial implantation. As part of the last operation, the rats were euthanized, and their femora were harvested. An illustration of the experimental setup is shown in Figure 1. The tissue samples were stored in an 8% formaldehyde solution in a freezer at −18 °C.

This experimental study was performed according to the German Laws on Animal Protection and was approved by the local animal care and use committee (Landesveterinär- und Lebensmitteluntersuchungsamt Mecklenburg-Vorpommern, Germany; reference/protocol number: 7221.3-1-055/16).

### 2.2. Osmotic Pump Implantation

The rats were anesthetized by intraperitoneally injecting ketamine hydrochloride 10% (100 mg per kg bodyweight, Bela-Pharm; Vechta, Germany) and xylazine hydrochloride 2% (25 mg per kg bodyweight, Rompun^®^, Bayer Vital; Leverkusen, Germany). While undergoing surgery, the rats were positioned in a prone position on a heating pad (Klaus Effenberger, Medizinische Geräte; Pfaffing, Germany) to counteract thermal dysregulation due to anesthesia. Postoperatively, oral analgesia was administered by adding metamizole to the drinking water (100 mg per kg bodyweight, Novaminsulfon-ratiopharm^®^, Ratiopharm; Ulm, Germany). From a posterior approach, a 2 cm skin incision between both medial scapula margins was performed, after shaving and sterilizing an area of 3 × 3 cm with povidone–iodine solution (Betaisodona^®^, Mundipharma GmbH; Frankfurt am Main, Germany). After blunt preparation, the osmotic pumps were implanted epifascially. The incision was closed intracutaneously (Vicryl^®^ 3-0, Johnson & Johnson; New Brunswick, NJ, USA) and disinfected again with the povidone–iodine solution. No wound infections were observed, and it was never necessary to widen the skin incision when exchanging the pump, since the pump could always be removed through the existing scar from the previous surgery. The osmotic pumps (5.1 cm length, 1.4 cm diameter; ALZET^®^ MODEL 2ML4; DURECT Corporation; Cupertino, CA, USA) were used for the continuous application of BPA and the control solution DMSO. The pump rate was 2.5 µL/h (standard deviation ± 0.05 µL/h). DMSO does not affect the rat bone, and it is frequently used in experimental studies with rats due to its generally good tolerability [20,26]. The interventions took 5 min each. As mentioned above, after 12 weeks, the final surgery was performed in which the rat femora were collected, and the rats were euthanized by anesthesia overdose, followed by breaking their neck.

### 2.3. Micro-Computed Tomography (Micro-CT)

Micro-CT analysis was used to visualize the structural properties of the cortical and trabecular bone. The scans were performed using a high-resolution device (µCT 35, Scanco Medical AG; Brüttisellen, Switzerland; 70 kVp, 114 µA, 400 ms integration time). The isotropic voxel dimension was related to the different sizes of the animals adapted to the sex: 12 × 12 × 12 µm for female and 20 × 20 × 20 µm for male rats. By using a constrained Gaussian filter (trabecular bone: support = 1, sigma = 0.8; cortical bone: support = 2, sigma = 0.8), grayscale CT images were segmented; accordingly, the background noise of the original data could be reduced. For assessment of trabecular bone, 75 (1.5 mm, males) or 125 (1.5 mm, females) slices were evaluated in the distal femur with the volume of interest 2.5 mm below the growth plate, which included only the secondary spongiosa. The structure of the cortical bone was analyzed at the mid diaphysis. In doing so, 1 mm of bone, resulting in 83 slices in female and 50 slices in male rats, was included. In order to extract the trabecular bone, data were globally thresholded at 26%. For the cortical bone, a threshold of 30% was used. For the trabecular bone, the following parameters were obtained: bone volume fraction (BV/TV), trabecular number (Tb.N, 1/mm), trabecular separation (Tb.Sp, mm), and trabecular thickness (Tb.Th, mm). For the cortical bone, the following parameters were obtained: total cross-sectional area inside the periosteal envelope (Tt.Area, mm^2^), cortical thickness (Ct.Th, mm), cortical area (Ct.area, mm^2^), bone marrow area (mm^2^) and the relation of the cortical area to total area (Ct.Ar/Tt.Ar, %). In addition, the length of the femora (mm) and the connectivity density (mm^−3^) were measured.

The guidelines for assessment of bone microstructure in rodents using micro-computed tomography by Bouxsein et al. were utilized for all of the analyses [27].

### 2.4. Biomechanical Testing

The servo-controlled electromechanical testing machine Z2.5/TN1S (ZwickRoell; Ulm, Germany) with a 2.5 kN load cell was used to analyze the biomechanical properties of the femoral rat bones by creating load–displacement and stress–strain curves until the breaking point. The two support points (4 mm diameter) on which the bone were 15 mm (females) or 20 mm (males) apart from each other, and the probe applied the force perpendicularly in the middle of the specimen in an anterior–posterior direction. The preloading was 0.1 N (0.1 mm/s), and the loading rate was 10 mm/min. The following parameters were obtained until the bones broke: ultimate load (N), ultimate displacement (mm), energy (mJ), energy density (mJ/mm^3^), stiffness (N/mm), ultimate stress (MPa), ultimate strain, and Young’s modulus (MPa). The Young’s modulus and the stiffness were defined by the slope of the linear part of the stress–strain and load–deformation curves, respectively. For the calculation of the stress–strain curve, peripheral computed tomography (pQCT, XCT Research SA, Stratec Medizintechnik; Pforzheim, Germany) was used to determine the area moment of inertia (mm^4^).

All analyses of the biomechanical testing were based on the guide “Basic Biomechanical Measurement of Bone: A Tutorial” by Turner and Burr [28].

### 2.5. Statistical Analysis

All results were evaluated with SPSS Statistics (Version 25; IBM, Armonk, NY, USA) At first, we analyzed the data with the D’Agostino Pearson normality test for normal distribution which did not show a consistent normal distribution for all of the values. Hence, a nonparametric Kruskal–Wallis test (H-test) was performed to determine whether the values, which were not normally distributed and considered to be independent samples, differed in terms of a central tendency. The advantage of the test is that the data do not have to be normally distributed [29]. Subsequently, a Mann-Whitney test was performed between each of the groups. This test is also valid when there is no normal distribution of the data [29]. No adjustment for multiple testing was performed. A value was considered to be significant when the *p*-value was less than 0.05. Tendencies were defined by *p*-values between 0.05 and 0.1.

## 3. Results

Two rats of the female high-dose group died during the first anesthesia; therefore, a total of 34 instead of 36 animals were included for further examination of the bones and statistical analysis.

When comparing the three different groups of each sex with the Kruskal–Wallis test, no significant differences were detected. The *p*-values refer to the Mann-Whitney U tests that were performed simultaneously. Significant differences were found when comparing the male and female control groups, in which only the control solution DMSO was administered. They differed significantly in terms of length of the femora (*p* = 0.004), bone volume fraction (*p* = 0.018), connectivity density (*p* = 0.004), trabecular number (*p* = 0.01), trabecular thickness (*p* = 0.025), trabecular separation (*p* = 0.01), total cross-sectional area inside the periosteal envelope (*p* = 0.004), cortical area (*p* = 0.004), and the bone marrow area (*p* = 0.004). The three-point bending test found significant differences regarding the area moment of inertia (*p* = 0.004), ultimate load (*p* = 0.01), ultimate displacement (*p* = 0.016), and the energy (*p* = 0.004) when comparing both control groups. Except for the bone volume fraction, the connectivity density, and the trabecular number, which were higher in the female control group, all other parameters were higher in the male control group.

**Figure 2:**
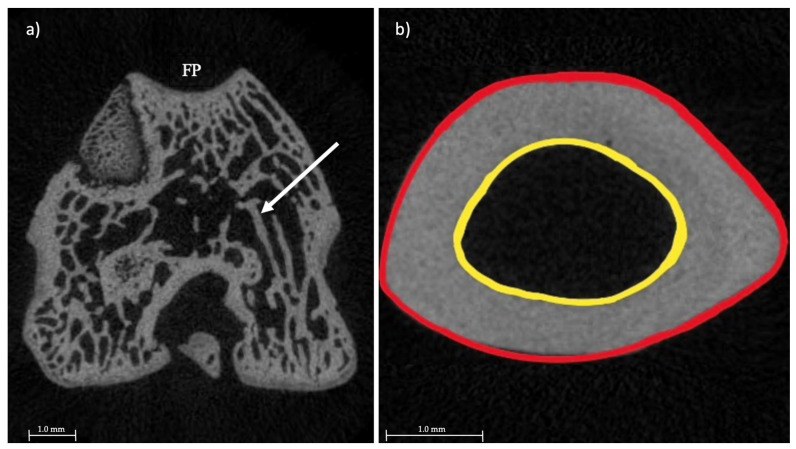
Cross-sectional micro-CT images. The left image (**a**) shows the femoral metaphysis with the white arrow pointing to one of the trabeculae; FP = facies patellaris femoris. The right image (**b**) pictures the femoral diaphysis. The area within the red circle is equal to the total cross-sectional area (Tt.Ar) and consists of the cortical area (Ct.Ar., area in between the red and yellow circle) and the marrow area (area within the yellow circle). The top of the figure is the anterior aspect, while the bottom of the figure is the posterior aspect of the bone.

Figure 2 shows cross-sectional micro-CT images of the femoral meta- and diaphysis which were used for the measurements of the beforementioned morphological parameters.

Since these clear differences were already apparent when comparing the male and female control groups, the comparison of male and female rats was limited in its significance when comparing both low-dose and high-dose groups with each other. Nevertheless, the control groups were crucial when estimating dose-related effects of BPA within the groups of male and female rats.

### 3.1. Micro-Computed Tomography

**Figure 3:**
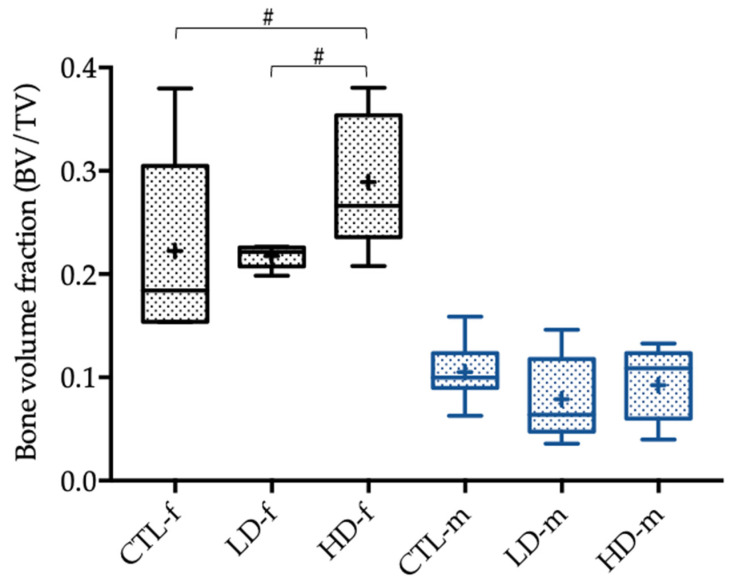
Bone volume fraction (BV/TV) of female (-f) and male rats (-m) 12 weeks after administration of the control solution (CTL; *n* = 6 CTL-f, *n* = 6 CTL-m), low-dose BPA (LD; *n* = 5 LD-f, *n* = 5 LD-f), and high-dose BPA (HD; *n* = 5 HD-f, *n* = 7 HD-m). The low-dose group was administered 10.2 µg BPA per kg bodyweight per day, and the high-dose group was administered 156 µg BPA per kg bodyweight per day. The data are visualized with boxplots; the mean is marked with a cross (+) and tendencies (*p* < 0.1, but ≥ 0.05) are marked with a pound (#). Differences between male and female rats are not marked.

Analyzing the bone morphology by micro-CT revealed several group-specific differences. The bone volume fraction is the relation of bone volume to the total tissue volume pictured in a cross-sectional micro-CT slide as a percentage. The bone volume fraction of the female control group was significantly higher when compared with the male control group (*p* = 0.01). We could show a tendency not only when comparing the female low-dose and high-dose groups (*p* = 0.076), but also when comparing the female high-dose and control groups (*p* = 0.076). The mean of the bone volume fraction was higher in the high-dose group in both cases (Figure 3).

Further statistical tendencies could be shown when comparing the female low-dose and high-dose groups with respect to the number of trabeculae (*p* = 0.076) and the total cross-sectional area inside the periosteal envelope (*p* = 0.076). However, the trabecular number and the total cross-sectional area inside the periosteal envelope were higher in the low-dose group.

In male rats, statistical tendencies could be shown by comparing the low-dose group with the high-dose group regarding the length of the femora (*p* = 0.062), the total cross-sectional area inside the periosteal envelope (*p* = 0.062), and the cortical area (*p* = 0.088). The femora were longer in the high-dose group but with lower values of the cortical area of the same femora.

**Figure 4:**
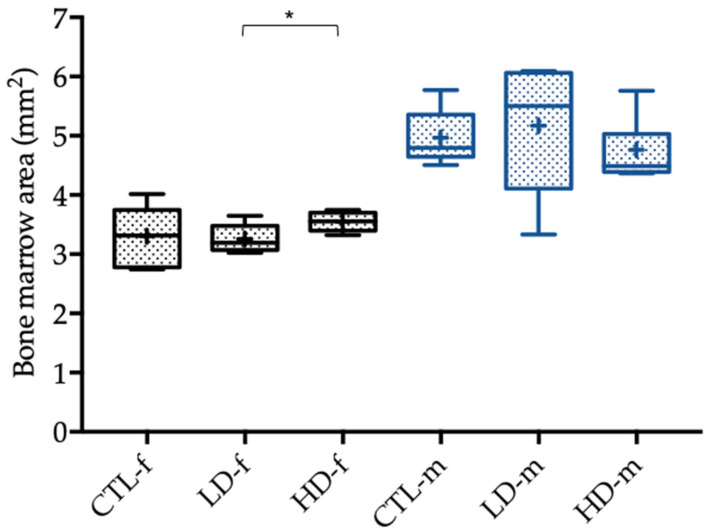
Cross-sectional bone marrow area of the femoral bone of female (-f) and male rats (-m) in mm^2^ 12 weeks after administration of the control solution (CTL; *n* = 6 CTL-f, *n* = 6 CTL-m), low-dose BPA (LD; *n* = 5 LD-f, *n* = 5 LD-f), and high-dose BPA (HD; *n* = 5 HD-f, *n* = 7 HD-m). The low-dose group was administered 10.2 µg BPA per kg bodyweight per day, and the high-dose group was administered 156 µg BPA per kg bodyweight per day. The data are visualized with boxplots; the mean is marked with a cross (+) and significant differences are marked with an asterisk (*). Differences between male and female rats are not marked.

Cross-sectional areas of the bone marrow were significantly higher in the female high-dose group than in the female low-dose group (*p* = 0.047, Figure 4).

### 3.2. Three-Point Bending Test

**Figure 5:**
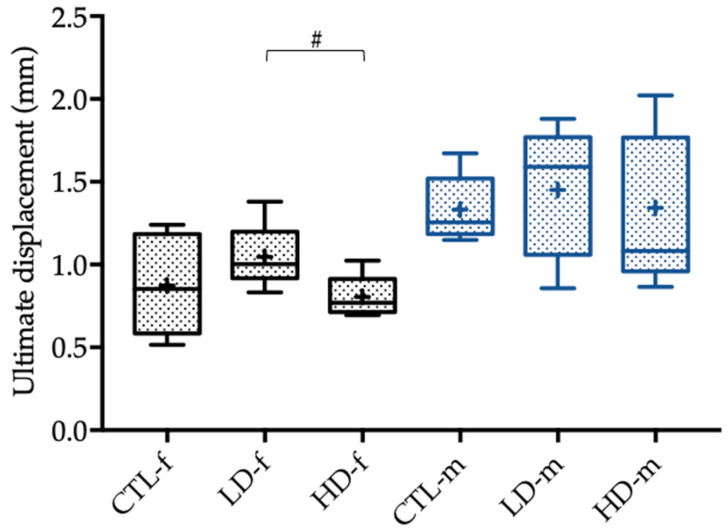
Ultimate displacement of female (-f) and male rats (-m) in mm 12 weeks after administration of the control solution (CTL; *n* = 6 CTL-f, *n* = 6 CTL-m), low-dose BPA (LD; *n* = 5 LD-f, *n* = 5 LD-f), and high-dose BPA (HD; *n* = 5 HD-f, *n* = 7 HD-m). The low-dose group was administered 10.2 µg BPA per kg bodyweight per day, and the high-dose group was administered 156 µg BPA per kg bodyweight per day. The data are visualized with boxplots; the mean is marked with a cross (+) and tendencies (*p* < 0.1, but ≥ 0.05) are marked with a pound (#). Differences between male and female rats are not marked.

**Figure 6:**
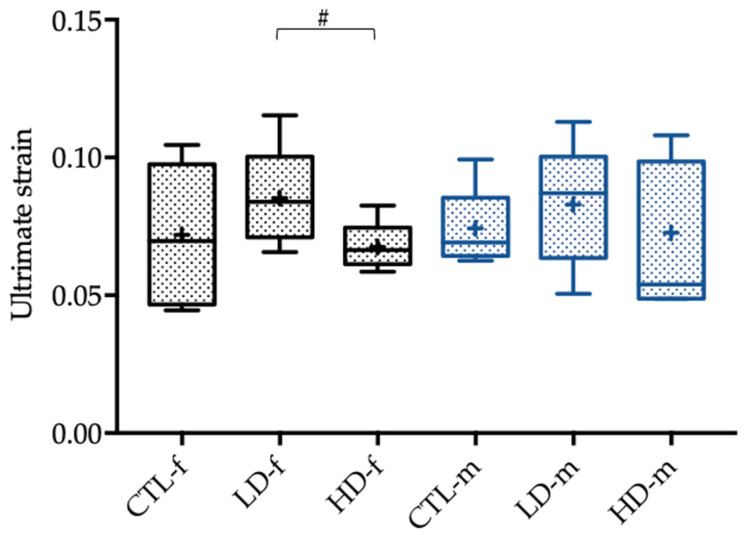
Ultimate strain of female (-f) and male rats (-m) 12 weeks after administration of the control solution (CTL; *n* = 6 CTL-f, *n* = 6 CTL-m), low-dose BPA (LD; *n* = 5 LD-f, *n* = 5 LD-f), and high-dose BPA (HD; *n* = 5 HD-f, *n* = 7 HD-m). The low-dose group was administered 10.2 µg BPA per kg bodyweight per day, and the high-dose group was administered 156 µg BPA per kg bodyweight per day. The data are visualized with boxplots; the mean is marked with a cross (+) and tendencies (*p* < 0.1, but ≥0.05) are marked with a pound (#). Differences between male and female rats are not marked.

The evaluation of the biomechanical properties of the female femoral rat bones showed tendencies toward a decrease in the ultimate displacement (*p* = 0.076), as well as the ultimate strain (*p* = 0.076), in those groups that were exposed to higher BPA concentrations (Figure 5 and Figure 6).

**Figure 7:**
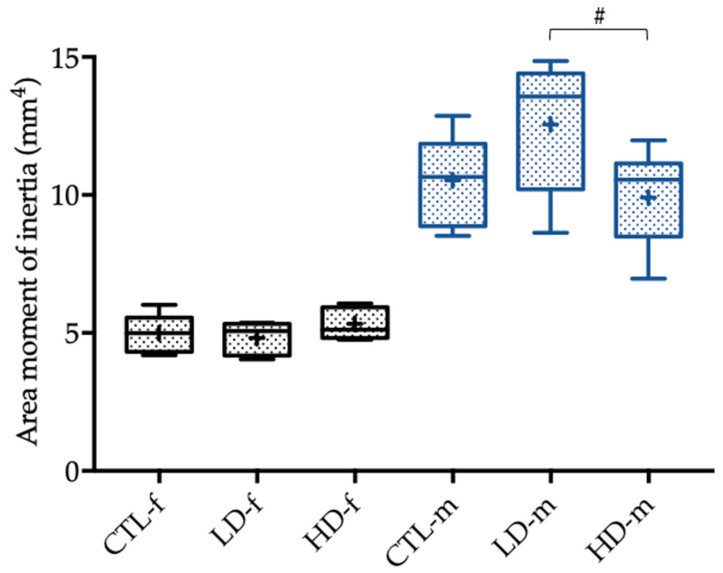
Area moment of inertia of female (-f) and male rats (-m) in mm^4^ 12 weeks after administration of the control solution (CTL; *n* = 6 CTL-f, *n* = 6 CTL-m), low-dose BPA (LD; *n* = 5 LD-f, *n* = 5 LD-f), and high-dose BPA (HD; *n* = 5 HD-f, *n* = 7 HD-m). The low-dose group was administered 10.2 µg BPA per kg bodyweight per day, and the high-dose group was administered 156 µg BPA per kg bodyweight per day. The data are visualized with boxplots; the mean is marked with a cross (+) and tendencies (*p* < 0.1, but ≥ 0.05) are marked with a pound (#). Differences between male and female rats are not marked.

Another tendency could be found between the male low-dose and high-dose groups regarding the area moment of inertia (*p* = 0.062), which was higher in the low-dose group (Figure 7).

## 4. Discussion

In this experimental study, 18 healthy male and 16 female rats were kept under controlled conditions for 12 weeks in an animal facility to reveal any effects of continuous intraperitoneal application of low and high dosages of bisphenol A on bone morphology and biomechanics.

The statistically significant differences in terms of the micro-CT analysis and the biomechanical properties between the male and female control groups described above were due to sex-specific differences in height and weight. This resulted in significantly larger bones in male rats and subsequently higher biomechanical load-bearing capacity shown in the three-point bending test and greater trabecular and cortical bone structures in micro-CT analysis. Considering the results and related tendencies, as well as the significant difference of the bone marrow area, when comparing female low-dose and high-dose groups, these differences were conspicuously seen more often in female rats. Additionally, the statistical tendencies were all found in between the low-dose and high-dose groups except for the bone volume fraction, where the high-dose and control group differed. In male rats, no statistically significant differences were found in relation to the control group, and the tendencies were all in between the low-dose and high-dose groups. The differences in between the micro-CT parameters, as seen in Figure 3 and Figure 4, were sex-specific and related not only to endogenous hormones but also to the body mass of the rats [27,30].

We could show a tendency regarding the bone volume fraction in the female high-dose group compared with the control group, as well as when comparing the female low-dose group with the high-dose group. In both cases, the proportion of the bone tissue with respect to the total tissue was higher in the group exposed to higher BPA concentrations. In addition, a greater number of trabeculae in the female high-dose group was found when compared to the low-dose group; hence, we assume that BPA might increase trabecular structures in rat bone. This was underlined by the tendency of a higher total cross-sectional area in the female high-dose groups when compared to the female low-dose group. Most importantly, the cross-sectional area of the bone marrow was significantly higher in the female high-dose group in comparison to the bone marrow area in the female low-dose group. This is similar to the findings of Lejonklou et al., who identified a significantly higher bone mineral content in female rats after high-dose exposure of BPA compared to low-dose exposure [31]. It also emphasizes that high-dose BPA exposure might rather have an anabolic effect on bone tissue which corroborates our hypothesis. Possible explanations for a significantly greater bone marrow area in the female high-dose group compared to the low-dose group could be due to BPA-induced thicker trabecular bone structures resulting in a greater marrow space, as well as a thicker cortical bone.

Lind et al. observed BPA-induced bone marrow fibrosis in female rats which might also be a reason for the significantly increased bone marrow area in our rats [32]. However, the cortical thickness and area did not show any tendency or a statistically significant increase in any of the groups. When cortical cross-sectional area alone was considered, no statistically significant difference was found when comparing the female cohorts.

In contrast, one tendency indicated a higher cross-sectional area inside the periosteal envelope in the male low-dose group when compared to the male high-dose group. Taking the BPA-related effects on female rat bone into account, a possible threshold of BPA could be discussed after which BPA was osteoinductive in female rats but led to a negative influence on bone metabolism after a certain concentration in male rats. Previously, this correlation could only be proven in part by other studies [17,31,32,33].

Tendencies in the biomechanical analyses could be shown in between the female low-dose and high-dose groups. Lower strain and lower ultimate displacement in the female high-dose group compared to the low-dose group indicated a possible higher resistance of the femora of the female low-dose group and subsequently a positive effect of low-dose BPA on bone stability. Tendencies of a higher total cross-sectional area, as well as cortical cross-sectional area and a higher femur length in the low-dose group, underlined this assumption.

According to a study by Miki et al., BPA affects human fetal and adult osteoblasts via steroid receptors [34]. Their study showed an activation of the cell proliferation rate and an accumulation of collagen in osteoblasts; thus, the authors hypothesized that BPA affects bone metabolism negatively in humans. Another study showed a reduced femur length and trabecular area in male offspring of pregnant rats who were exposed to low-dose BPA concentrations in their drinking water [33]. This conflicts with the observations made in our experiment when comparing the male low-dose and high-dose groups. After re-evaluation of these results with 52-week-old rats, the male cohort that was prenatally exposed to increased levels of BPA showed a normalization of the parameters collected to assess the bone structure and were comparable to the control group [32]. Lejonklou et al. demonstrated elongated rat femora in the female offspring of pregnant rats exposed to BPA via tube feeding and thicker cortical bone in the male offspring with no significant change of biomechanical parameters [31]. When analyzing our data, numerous tendencies could be shown in the comparison of the low- and high-dose groups within the male and female groups. Our results are mostly based on tendencies that indicate an osteoinductive effect of BPA after high-dose exposure in the female cohort, as well as a negative effect on bone metabolism after high-dose exposure in male rats only. This partially corroborates our hypothesis and the findings of Lind et al. described above [32,33]. Nevertheless, these findings are mostly based on weak tendencies between two of the three groups of each sex.

Discussing the methods of our study, the subcutaneous application of BPA by pumps working with osmosis via diffusion of molecules through a semipermeable membrane represented a reliable procedure [35,36]. The amount of BPA in the high-dose group was based on adverse effects of BPA that other studies found when administering the same or lower BPA dosages [37]. Adverse effects include toxicity on the reproductive system [38,39], as well as the estrogen-mimicking and endocrine-disrupting effect of BPA [17]. Focusing on the advantages of the micro-CT, it seemed to be favorable compared to procedures addressing histomorphology only as it created a 3D reconstruction of trabecular bone structures. Accordingly, the evaluation of trabecular bone structures was not based on previously obtained two-dimensional data. Micro-CT imaging is also advantageous due to the large area that can be analyzed, the speed of the analysis, and the fact that the objects are not damaged by imaging them [27,40]. In addition to micro-CT imaging, we used the three-point bending test to detect BPA-related changes in the rat bone. Both methods can be used to reliably assess the microarchitecture, as well as the biomechanical properties, of bones. However, they cannot provide information about cellular processes or the bone metabolism on a molecular level.

One weakness of our study was the sex-related difference in terms of size and weight and the associated different measurement results in the biomechanical tests, as well as the micro-CT examination. As a result, it was not possible to sum up the four male and female BPA-exposed groups as a whole to one low-dose and one high-dose group only. Furthermore, even if osmotic pumps deliver drugs reliably, they cannot provide continuous dosages per kilogram bodyweight, because the rats gain weight over time which would require an increasing dosage with the natural increase in bodyweight. Daily bodyweight-dependent dosages could be realized by subcutaneous injections in future studies. Furthermore, due to sex-specific weight differences at the very beginning of our experiments, male rats were 14% heavier than the females (302 g vs. 259 g); hence, the BPA dose per kilogram bodyweight was higher in female rats than in the male cohort. One should also take into account that the BPA-mediated effects on the bone are not linear [17]. The dosages of daily applied BPA in other studies varied from 0.25 to 50,000 µg per kilogram bodyweight. The heterogeneity of former study designs makes it harder to compare them with our findings [31,33]. As a result, inconsistencies are more likely to occur. Additionally, adding a mid-dose group might have been advantageous to further elucidate the dose-dependent effect of BPA. Furthermore, we focused on certain bone properties in terms of micro-computer tomographic morphology and biomechanics only. We found a significantly greater bone marrow area in female rats after high-dose BPA exposure when compared to the female low-dose group, which could indicate a possible osteoanabolic effect of BPA in female rats. In the same group, the trabecular number and the bone volume fraction were also higher, underlining this hypothesis. Nevertheless, further studies are needed to provide additional data since our results did only show tendencies except for one significant difference regarding the bone marrow area of the female cohort.

## 5. Conclusions

BPA is a ubiquitously used chemical with various adverse effects not only on rodents but also on human metabolism and the human hormonal system. We found an increase in the bone marrow area after high-dose exposure to BPA in female rats, as well as nonsignificant tendencies of an increase in the number of trabeculae and bone volume fraction when performing micro-CT analysis. In male animals, only a negative tendency of a change in bone morphology could be identified. Considering our data, the effects of BPA on the biomechanical properties and morphological changes quantified by micro-CT were limited. Further and longer-lasting studies are needed to analyze the molecular interaction of different dosages of BPA with bone to clarify BPA-related long-term effects.

## Data Availability

The data that support the findings of this study are available from the corresponding author, T.P., upon reasonable request.
